# Neighbourhood walkability and dietary attributes: effect modification by area-level socio-economic status

**DOI:** 10.1017/S1368980022001197

**Published:** 2022-09

**Authors:** Manoj Chandrabose, Yingting (Tina) Cao, Nyssa Hadgraft, Carl Higgs, Faysal Shuvo, David W Dunstan, Neville Owen, Takemi Sugiyama

**Affiliations:** 1 Centre for Urban Transitions, Swinburne University of Technology, Hawthorn, VIC 3122, Australia; 2 Baker Heart and Diabetes Institute, Melbourne, Australia; 3 Nossal Institute for Global Health, Melbourne School of Population and Global Health, University of Melbourne, Melbourne, Australia; 4 Centre for Urban Research, RMIT University, Melbourne, Australia; 5 Mary MacKillop Institute for Health Research, Australian Catholic University, Melbourne, Australia

**Keywords:** Built environment, Population health, Health inequalities, FFQ, Cardiometabolic diseases

## Abstract

**Objective:**

Higher neighbourhood walkability would be expected to contribute to better health, but the relevant evidence is inconsistent. This may be because residents’ dietary attributes, which vary with socio-economic status (SES) and influence their health, can be related to walkability. We examined associations of walkability with dietary attributes and potential effect modification by area-level SES.

**Design:**

The exposure variable of this cross-sectional study was neighbourhood walkability, calculated using residential density, intersection density and destination density within 1-km street-network buffer around each participant’s residence. The outcome variables were dietary patterns (Western, prudent and mixed) and total dietary energy intake, derived from a FFQ. Main and interaction effects with area-level SES were estimated using two-level linear regression models.

**Setting:**

Participants were from all states and territories in Australia.

**Participants:**

The analytical sample included 3590 participants (54 % women, age range 34 to 86).

**Results:**

Walkability was not associated with dietary attributes in the whole sample. However, we found interaction effects of walkability and area-level SES on Western diet scores (*P* < 0·001) and total energy intake (*P* = 0·012). In low SES areas, higher walkability was associated with higher Western dietary patterns (*P* = 0·062) and higher total energy intake (*P* = 0·066). In high SES areas, higher walkability was associated with lower Western diet scores (*P* = 0·021) and lower total energy intake (*P* = 0·058).

**Conclusions:**

Higher walkability may not be necessarily conducive to better health in socio-economically disadvantaged areas. Public health initiatives to enhance neighbourhood walkability need to consider food environments and socio-economic contexts.

The burden of cardiometabolic diseases (e.g. type 2 diabetes, heart disease and stroke) continues to increase globally^(1)^. Population-wide approaches are advocated as effective and sustainable strategies to combat the rise in cardiometabolic diseases and their risk factors^(2)^. Public health initiatives focusing on the built environment are important in this regard, as the features of neighbourhood environments are known to be associated with residents’ health-related behaviours, such as physical activity^(3)^.

There is a body of evidence supporting the relationships of neighbourhood walkability (a measure consisting typically of residential density, street connectivity and land use diversity) with walking for transport^(4)^ and physical activity^(5)^. Given the well-established health benefits of physical activity^(6,7)^, it can be expected that residents living in high-walkability neighbourhoods could have better cardiometabolic health profiles than those in lower walkability areas. However, there is inconsistent evidence for the relationships between walkability and cardiometabolic health indicators. Although a recent review of longitudinal studies reported evidence for the relationship between higher walkability and lower cardiometabolic risk^(8)^, an earlier review of mostly cross-sectional studies reported mixed findings on walkability and obesity^(9)^. More recent studies also identified null associations^(10)^, associations in an unexpected direction^(11,12)^ and non-linear associations between walkability and cardiometabolic health indicators^(13)^.

One potential explanation for this lack of consistency is that high-walkability neighbourhoods may have other environmental features that could influence health-related risk behaviours. For instance, high-walkability neighbourhoods, which have more commercial destinations, can have a wider range of food outlets, including those providing unhealthy food^(14,15)^. It has been shown that the perceived availability of unhealthy food is associated with poor dietary behaviours^(16,17)^. However, evidence on associations between neighbourhood walkability and residents’ dietary attributes is sparse, with only one study reporting that Walk Score^®^ (a web-based measure of neighbourhood walkability) was not associated with the consumption of vegetables and fruits among Taiwanese older adults^(18)^.

Area-level socio-economic status (SES) is also known to be related to residents’ dietary attributes^(19,20)^. A recent systematic review of Australian studies found that socio-economically disadvantaged areas tend to have more unhealthy food outlets^(21)^, suggesting a possibility that high-walkability low-SES neighbourhoods may have greater availability of unhealthy food options. It can be thus postulated that walkability and area-level SES may be jointly associated with residents’ dietary attributes. For instance, residents of high-walkability low-SES neighbourhoods may have poor dietary attributes due to the exposure to unhealthy food outlets.

In this cross-sectional study, we first examined associations of neighbourhood walkability with residents’ dietary attributes, then examined whether such associations may be moderated by area-level SES.

## Methods

### Data source and study participants

We used data from the third wave of the Australian Diabetes, Obesity and Lifestyle Study (AusDiab3), which collected data in 2011–2012. Details of this study have been described previously^(22)^. Briefly, at baseline (1999–2000), participants were recruited from forty-two study sites located in the six Australian states and the Northern Territory (six sites in each), using a two-stage stratified cluster sampling method. Eligible participants were non-institutionalised adults aged over 25 years, without any physical/intellectual disabilities, and residing in private dwellings for at least six months prior to the data collection. At baseline, 11 247 participants (response rate: 55·3 %) provided data. Of these, 4614 (follow-up rate: 44·6 %) provided data in AusDiab3. The analytical sample size was 3590, after excluding seven participants who reported being pregnant during data collection (since pregnancy-related factors may influence dietary attributes), 133 whose residential locations could not be geocoded and 884 with missing data for outcome and potential confounding variables used in the study. Nearly half of AusDiab3 participants had relocated their residence after the baseline data collection, resulting in scattered residential locations across multiple regions in Australia.

### Exposure variable

The exposure variable was the neighbourhood walkability index, which was a composite measure of residential density, intersection density and destination density. The rationale, methods and spatial data sources used to calculate this walkability index are described in Supplemental Material 1. Briefly, it was calculated for each participant within a 1-km street-network buffer (sausage type) around the residential location. A 1-km distance was chosen to represent the local neighbourhood, as it has been shown to be a typical distance within which most home-based walking activities can take place^(23)^. Residential density was calculated as the total count of dwellings within the buffer divided by its area. Intersection density was calculated as the number of 4-way (or more) intersections within the buffer divided by its area. We used the density of 4-way (or more) intersections because it was observed as a better measure of street connectivity in the Australian context than the density of typically used 3-way (or more) intersections (see online supplementary material, Supplemental Fig. S1). Destination density was calculated as the total count of destinations to which residents would travel on a regular basis (supermarkets, convenience stores and public transport stops) within the buffer divided by its area. The unit for all density measures was counts/km^2^. The walkability index was expressed as a standardised score of the summed z-scores of each density measure.

### Outcome variables

The dietary attributes examined as the outcome variables were three dietary patterns and total dietary energy intake. Participants completed a validated self-administered semi-quantitative FFQ, which assessed the daily intake of seventy-four food items (on a ten-point frequency scale) over the past 12 months, with additional questions on usual eating habits and portion size^(24)^. Dietary patterns were identified by applying factor analysis to the estimated daily intake of these food items. The methods for identifying dietary patterns have been reported previously^(25)^. A brief overview of these methods is provided in Supplemental Material 2. Three dietary patterns were identified: Western diet, prudent diet and mixed diet. Western diet was characterised by high consumption of take-away foods, snacks, processed meat and red meat. Prudent diet was characterised by high consumption of vegetables (root, leafy and stalk) and fruits. Mixed diet was characterised by high consumption of fish, cereals, pasta, rice and poultry. For ease of interpretation, the three dietary factor scores (mean = 0, sd = 1) were transformed to have a mean of 100 with an sd of 25 (i.e. transformed value = original value × 25 + 100). The average daily intake of each food item (in grams) was converted into total dietary energy intake in kJ/d.

### Potential effect modifier

We examined area-level SES as a potential effect modifier of the relationships between walkability and dietary attributes. We used the Index of Relative Socio-economic Disadvantage (IRSD), which is a census-based composite index indicating the level of disadvantage of an area^(26)^. The Australian Bureau of Statistics calculates IRSD scores by applying principal component analysis to relevant area-level variables such as proportions of lower income households, unemployed people, households without cars, people with lower education and overcrowded private dwellings. Lower IRSD scores indicate more socio-economically disadvantaged areas. In the present study, we used the 2011 IRSD scores corresponding to Local Government Areas (LGA)^(27)^. LGA is an administrative division below State and Territory governments; there were 568 LGA in Australia in 2011. Each LGA is administered by a local council that is responsible for providing a range of services and infrastructure for the community. Participants in the current study resided in 196 LGA, which had a median population of 72 972 (first quartile: 36 506, third quartile: 152 389).

### Statistical analyses

Descriptive statistics for all variables were calculated for the whole sample and subgroups stratified by IRSD tertiles (low, medium and high SES areas).

For estimating the main effects (i.e. associations of neighbourhood walkability with dietary attributes), we used two-level random intercept linear regression models that accounted for potential area-level clustering (participants at level 1 and LGA at level 2)^(28)^. In the primary analyses, regression models adjusted for potential confounders: individual socio-demographic characteristics (gender, age, education, marital status, employment status, household income and children in the household) and area-level SES (IRSD).

To examine the potential effect modification by area-level SES on the association between walkability and dietary attributes, a two-way interaction term of walkability and IRSD was added to the regression model of each outcome and observed for an interaction effect. If an interaction was found, associations between walkability and dietary attributes were estimated for each subgroup stratified by IRSD tertiles.

All statistical analyses were conducted in R 4.0.5 (R Core Team). The package ‘lme4’ version 1.1.14 was used to model linear mixed models. The package ‘ggmap’ version 2·7·9 was used to geocode study participants’ residential addresses. For all other geographic information systems analyses, we used ArcGIS Pro version 2·3·3 (ESRI Inc).

## Results

Table 1 shows the key characteristics of study participants. Their mean age was 59 years (range: 34–86 years) and 54 % were women. The medium SES areas had a relatively higher mean walkability value compared with the low and high SES areas. The average total dietary energy intake was 7150 kJ/d.

**Table 1:** Characteristics of the analytical sample

	Low SES areas (*n* 1197)			Medium SES areas (*n* 1197)			High SES areas (*n* 1196)			Total (*n* 3590)		
	%	Mean	sd	%	Mean	sd	%	Mean	sd	%	Mean	sd
Age, years		58·8	10·0		58·8	9·9		59·1	9·7		58·9	9·9
Gender, % women	55			54			53			54		
Education,												
% High school or less	38			31			25			31		
% Technical/Vocational	47			45			41			44		
% Bachelor’s degree or more	16			24			35			25		
Employment status, % working	58			55			63			59		
Household income,												
% > $1500 per week	36			47			52			45		
% $600–$1500 per week	38			37			36			37		
% < $600 per week	26			16			12			18		
Marital status, % couple	78			77			83			79		
Children in the household, % yes	29			32			40			34		
Residential density (counts/km^2^)		513	284		870	574		649	423		677	467
Intersection density (counts/km^2^)		4·0	3·9		5·4	6·0		4·5	6·8		4·6	5·7
Destination density (counts/km^2^)		1·3	1·5		1·8	2·2		1·3	1·8		1·5	1·89
Walkability		−0·23	0·64		0·27	1·23		−0·10	1·06		−0·02	1·03
IRSD		955	27		1020	15		1060	21		1010	50
Western diet score		102·0	25·2		99·2	25·8		98·7	23·8		100·0	25·0
Prudent diet score		101·0	25·5		97·9	24·1		101·0	25·3		100·0	25·0
Mixed diet score		97·8	25·0		102·0	26·8		100·0	22·9		100·0	25·0
Total dietary energy intake (kJ/d)		7190	2830		7040	2770		7210	2710		7150	2770

SES, socio-economic status; IRSD, Index of Relative Socioeconomic Disadvantage corresponding to the Local Government Area where participant resided, a score of 1000 equals the national average, with lower scores indicating more socio-economically disadvantaged areas

Table 2 shows the results of the regression models fitted to estimate the main and interaction effects. As shown in the main effect models, the walkability index was not associated with any of the dietary attributes in the whole sample. However, we found interaction effects of walkability and IRSD on Western diet scores (*P* < 0·001) and total energy intake (*P* = 0·012), indicating that the associations of walkability with these two dietary attributes may vary by area-level SES. Such interaction effects of walkability and IRSD were not found for prudent and mixed diet scores.

**Table 2:** Associations of neighbourhood walkability with dietary attributes, AusDiab3 study, 2011–2012 (*n* 3590)

Dietary attributes	Main effect model			Interaction effect model		
	Walkability*			Walkability × IRSD		
	*β*	95 % CI	*P*	*β*	95 % CI	*P*
Western diet score	−0·08	−0·81, 0·64	0·818	−0·03	−0·05, –0·01	< 0·001
Prudent diet score	−0·01	−0·88, 0·86	0·979	0·00	−0·02, 0·03	0·711
Mixed diet score	0·21	−0·64, 1·07	0·624	0·00	−0·02, 0·03	0·715
Total dietary energy intake (kJ/d)	−20·10	−106·14, 65·93	0·647	−2·84	−5·05, –0·62	0·012

IRSD, Index of Relative Socioeconomic Disadvantage

* Regression coefficients correspond to one sd increment in walkability index. These models adjusted for age, gender, education, work status, household income, marital status, household children status, area-level socio-economic status (IRSD as a continuous variable) and corrected for area-level clustering. The interaction effect models additionally included the product of walkability and IRSD scores.

**Fig. 1:**
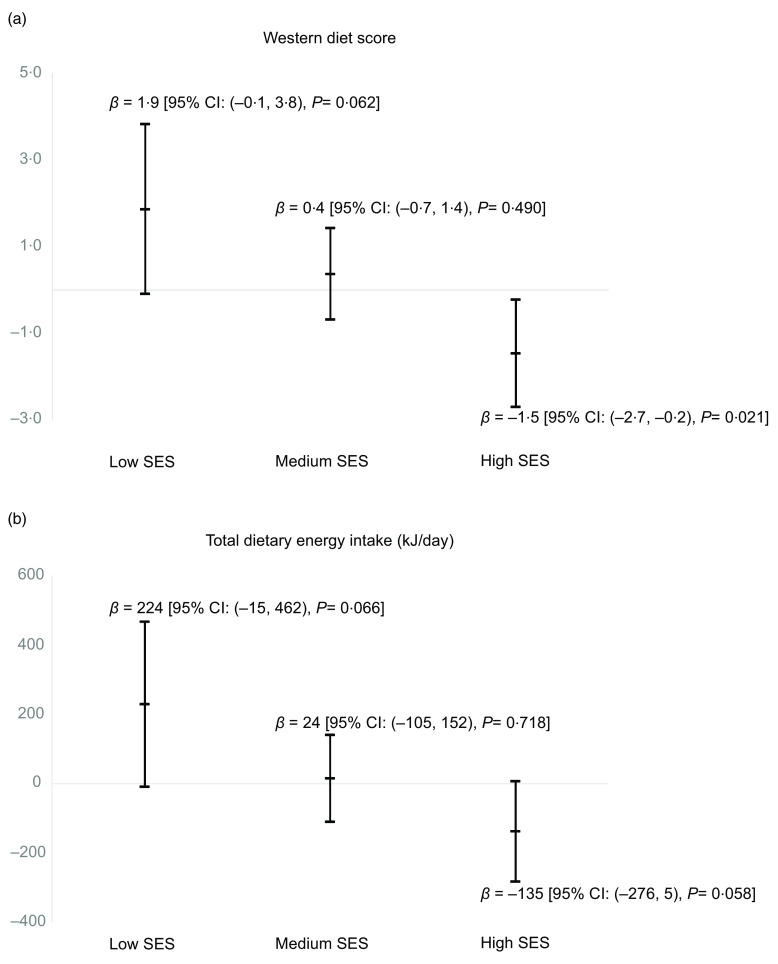
Regression coefficients for the Western diet score (a) and total dietary energy intake (b) corresponding to one sd increment in walkability index, stratified by area-level socio-economic status (SES), AusDiab3 study, 2011–2012 (*n* 3590). Each model was adjusted for age, gender, education, work status, household income, marital status, household children status and corrected for area-level clustering

As shown in Fig. 1, in low SES areas, walkability was positively associated with Western diet scores (*P* = 0·062) and total energy intake (*P* = 0·066). On the other hand, in high SES areas, walkability was inversely associated with Western diet scores *(P* = 0·021) and total energy intake (*P* = 0·058).

## Discussion

In our examination of the associations of neighbourhood walkability with dietary attributes using a cohort of Australian adults, we did not observe associations between walkability and any of the dietary attributes in the whole sample. However, we found interaction effects of walkability and area-level SES on Western dietary pattern and total energy intake. In the analyses stratified by area-level SES subgroups, higher walkability was associated with higher Western diet scores (i.e. higher consumption of take-away foods, snacks, red meat and processed meat) and higher total energy intake in low SES areas. In contrast, in high SES areas, higher walkability was associated with lower Western diet scores and lower total energy intake.

There is little evidence available on the associations between walkability and dietary attributes. Consistent with our main effect results, one Taiwanese study also did not find an association between neighbourhood walkability and consumption of vegetables and fruits among older adults^(18)^. However, this Taiwanese study is not directly comparable to our study because it was conducted in a non-Western setting, investigated older adults, used different types of exposure and outcome measures and did not examine the effect modification by area-level SES. Our findings contribute to advancing the understanding of the relationship between walkability and dietary attributes, by showing its dependence on the area-level SES.

Differential associations of walkability with dietary attributes observed between the low and high SES areas may be explained by differences in food environments, differences in individuals’ food preferences, or both. Previous research has demonstrated that high-walkability neighbourhoods have better access to more and diverse retail food outlets^(14,15)^. Further, Australian studies have consistently found that socio-economically disadvantaged areas tend to have a greater number of unhealthy food outlets^(21)^. In the context of our study, residents of high walkability neighbourhoods in low SES areas might have had greater exposure to unhealthy food outlets, whereas those residing in high walkability neighbourhoods in high SES areas might have better access to healthy food outlets. Given that availability of food types in local areas could be a key determinant of dietary attributes^(29,30)^, differential characteristics of neighbourhood food environments may be contributing to the observed moderation effect by area-level SES. In addition, differences in residents’ food preferences between low and high SES areas might also be contributing to the current findings^(29,30)^. Some population groups may prefer to purchase and consume unhealthy food due to affordability, ease of preparation, cultural background or lack of nutritional awareness^(29)^. It is possible that residents of high-walkability neighbourhoods in socio-economically disadvantaged areas may have chosen unhealthy foods for various reasons, even if healthy food options are available. Future research might consider investigating how these factors (food environments and food choice) are involved in the link between walkability, area-level SES and dietary attributes.

Research on the relationship between neighbourhood walkability and cardiometabolic health indicators (e.g. obesity, hypertension, type 2 diabetes, CVD and their risk markers) typically considers physical activity as the direct behavioural pathway^(8)^. Our findings suggest that dietary attributes may also be involved in this relationship. Higher consumption of unhealthy foods among residents of high walkability neighbourhoods in socio-economically disadvantaged areas may have partly contributed to the inconsistent findings observed for the associations between walkability and cardiometabolic health indicators^(10–13)^. In addition, high-walkability neighbourhoods may also expose residents to other environmental factors that can increase the risk of cardiometabolic diseases such as high levels of noise/air pollution, low levels of natural features (green spaces, blue spaces and tree cover) and psychological stress due to overcrowding^(31)^. Further empirical research needs to investigate how these environmental exposures are independently and jointly related to health behaviours and outcomes to obtain a more-comprehensive understanding of the relationship between the built environment and population health.

Our findings are relevant from a perspective of socio-economic inequalities in health. It can be argued based on the findings of this study and previous studies that higher walkability may benefit high SES areas due to greater physical activity and better dietary attributes, while such benefits may be limited in low SES areas. This could suggest that improving walkability (i.e. densifying areas through infill development and high-density redevelopment) may result in widening health disparities, if they are effective in promoting health-enhancing behaviours (more physical activity, healthier diet) only in high SES areas. Inequalities in health are persistent in our society and reducing them is a key challenge in public health^(32)^. To address this challenge, research may need to consider developing specific environmental initiatives targeting socio-economically disadvantaged neighbourhoods to reduce inequalities in health. Such focused efforts are warranted to prevent health disparities from worsening.

Our study has several strengths. We investigated a large sample of adults whose residences were in diverse geographical settings across Australia. This provided greater variabilities in walkability and area-level SES measures. We used a nationally consistent objective measure of neighbourhood walkability. In addition to total dietary energy intake, which is an important dietary attribute from an energy balance point of view for obesity prevention^(33)^, we also examined dietary patterns, which were characterised by not only the quantities of food intake but also by the patterns of specific types of food consumed. Such dietary pattern scores are considered to be better predictors of cardiometabolic disease risk^(34)^. A limitation of this study is the generalisability of the findings to the Australian population. There may have been a selection bias due to the relatively high attrition rate (55 %) at the 12-year follow-up. The average amount of total dietary energy intake in our study sample was slightly lower than the national average at that time^(35)^, which may be due to the attrition bias. Since this is a cross-sectional study, it is not possible to rule out the possibility of reverse causation, i.e. people with specific dietary attributes may have chosen to live in areas with better availability of their preferred food, rather than characteristics of the neighbourhood affecting their dietary attributes. Future quasi-experimental studies may examine whether moving to high walkability neighbourhoods changes dietary attributes and whether such changes are moderated by area-level SES.

## Conclusions

Our findings suggest that the relationships between neighbourhood walkability and residents’ dietary attributes are complex and subject to area-level socio-economic status. Urban planning initiatives being implemented to improve local area walkability, such as the ‘20-minute neighbourhoods’ development plan in Australia^(36)^, may assist residents to be more active in their neighbourhoods. However, such development plans may have differential health impacts on neighbourhoods with different levels of socio-economic status. If they provide a greater health benefit to only high SES areas, they can result in expanding health disparities. Our findings demonstrate the need to consider the combination of physical activity and dietary attributes with the socio-economic context where they take place in order to better understand the relationships between the built environment and health. Evidence from such research can contribute to developing more responsible urban planning policies that can enhance population health through multiple health behaviours without widening socio-economic inequalities in health.

## Supplementary material

For supplementary material accompanying this paper visit https://doi.org/10.1017/S1368980022001197. click here to view supplementary material
