# Supplementary material for: Neighbourhood walkability and dietary attributes: effect modification by area-level socio-economic status
Source: Public Health Nutr. 2022 May 18;25(9):2593–600. doi: 10.1017/S1368980022001197 (PMC9991640; doi:10.1017/S1368980022001197)
Supplement: Supplementary file 1 [file S1368980022001197sup.zip › S1368980022001197sup001.docx]

# Supplementary Material 1: Calculating neighbourhood walkability index

The neighbourhood walkability index typically consists of measures of residential density, street connectivity, and land use mix. Given the difficulty of obtaining Australia-wide fine-scale land use data, it was recommended to use destination density as an alternative measure (instead of the land use mix entropy measure) for calculating the walkability index (Mavoa *et al*. 2018). Measurement methods for each of these components are described below. We calculated the walkability index for each participant within a 1-km distance street-network buffer (sausage-type, with 150m radius from the street centre line) around their residential location. Such a buffer contains the area that can be reached within a 1-km distance along pedestrian accessible roads from the home with a 150m radius bandwidth from the street centre line. The road network data from the Public Sector Mapping Agency (PSMA) Transport & Topography dataset (PSMA Australia, 2012) was used for creating street network buffers.

Residential density

Residential density was defined as the number of dwellings in the buffer divided by its area. The Australian 2011 Census dwelling count data (Australian Bureau of Statistics, 2011) were used to calculate this variable. The Mesh Block (MB) was the smallest building block for which dwellings count data were available. The total dwellings count for a buffer was calculated by summing the respective counts of the MB units included in the buffer. If the buffer intersected an MB unit, then that unit’s count corresponding to the percentage of the area within the buffer was included.

Intersection density

We used intersection density as the measure of street connectivity. This was defined as the number of 4-way (or more) intersections within the buffer divided by its area. The road network data from PSMA Transport & Topography dataset (PSMA Australia, 2012) was used to calculate intersection density. In general, 3-way (or more) intersection density is used as a measure of street connectivity to calculate walkability (Frank *et al.,* 2010). However, in the geographical context of the current study, it was observed that street-network buffers with a higher number of 3-way (or more) intersections have more dead-end streets and cul-de-sacs, which contribute to lower walkability.

**Buffer A**

| 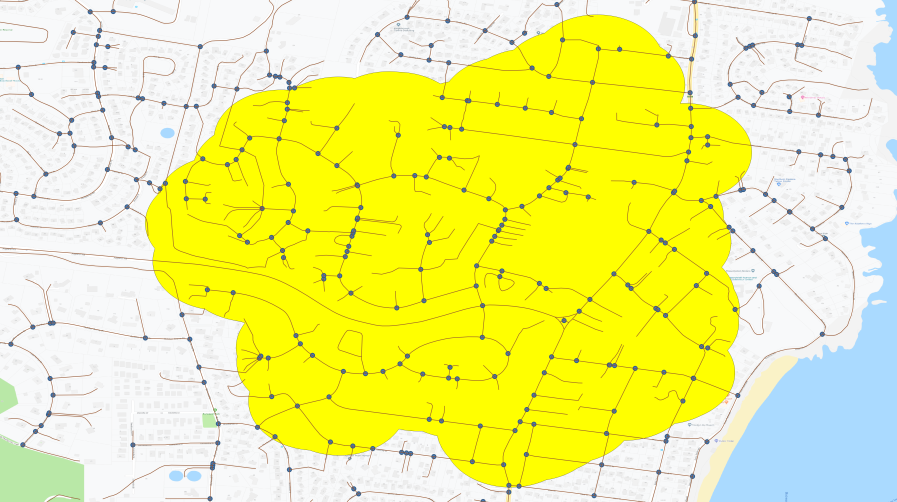  **3-way (or more) intersections = 130 counts / km^2^** | 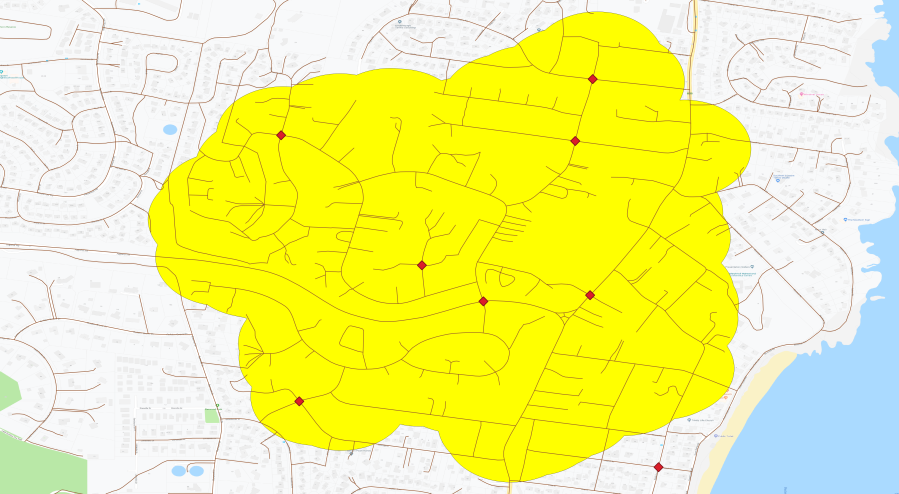  **4-way (or more) intersections = 5 counts / km^2^** |
| --- | --- |

**Buffer B**

| 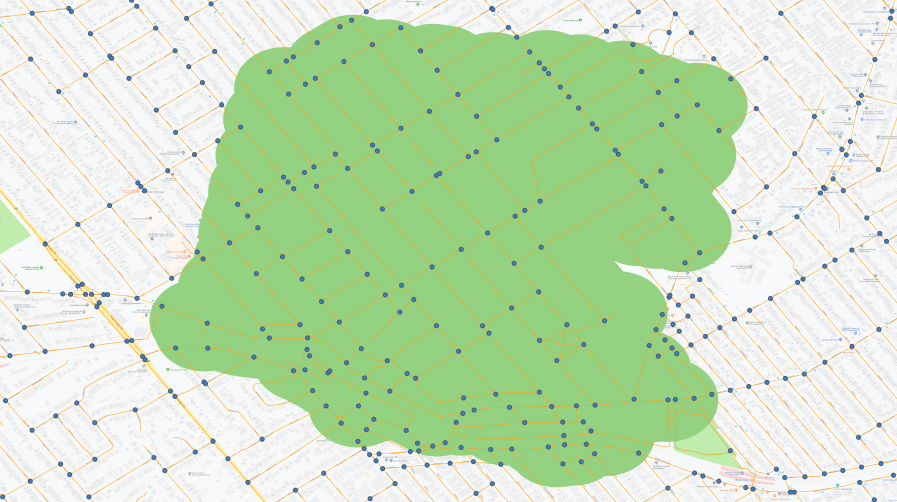  **3-way (or more) intersections = 77 counts / km^2^** | 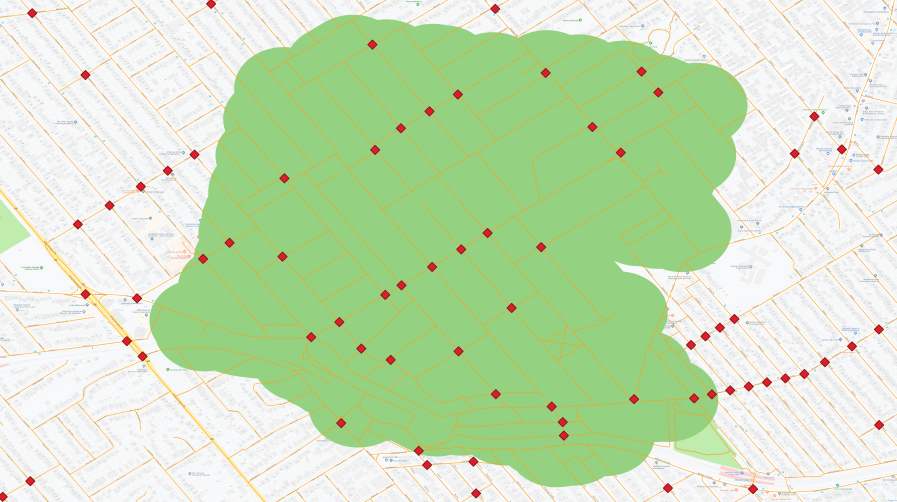  **4-way (or more) intersections = 15 counts / km^2^** |
| --- | --- |

**Figure S1: A comparison of two intersection density measures**

For instance, as shown in Figure S1, Buffer A has a higher density of 3-way (or more) intersections than Buffer B. But, many of those intersections in Buffer A are dead-end streets and cul-de-sacs. On the other hand, Buffer A has a lower density of 4-way (or more) intersections than Buffer B. Given that the density of 4-way (or more) intersections has previously been shown to be associated with walking in the context of Australia (Turrell, 2013), we decided to use it as an appropriate measure of street connectivity to calculate the walkability index.

Destination density

Destination density was defined as the number of supermarkets, convenience stores, and public transport stops within the buffer divided by its area. These destinations are considered as local places to which residents may travel regularly. Axiom Business Points data and Supermarkets data, sourced in 2012–2013, (Pitney Bowes Ltd, 2014) were used to obtain locations of convenience stores and supermarkets. PSMA Transport & Topography dataset (PSMA Australia, 2012) and the General Transit Feed Specification online repository data (<http://transitfeeds.com>, sourced in 2015) were used to obtain locations of public transport stops.

Reference:

Australian Bureau of Statistics (2011) *Census of population and housing: mesh block counts, 2011 (cat. no. 2074)* Canberra, Australia.

Frank LD, Sallis JF, Saelens BE *et al.* (2010) The development of a walkability index: application to the Neighborhood Quality of Life Study. *Br J Sports Med* 44, 924-933.

Mavoa S, Eagleson S, Badland HM *et al.* (2018) Identifying appropriate land-use mix measures for use in a national walkability index. *Journal of Transport and Land Use* 11.

Pitney Bowes Ltd. (2014) Axiom business points – 2014 release. *Availble at:* <https://www.pitneybowes.com/au>

PSMA Australia. (2012) Transport & Topography - 2012 release. *Availble at:* [*https://portal.aurin.org.au/*](https://portal.aurin.org.au/)

Turrell G, Haynes M, Wilson L-A *et al.* (2013) Can the built environment reduce health inequalities? A study of neighbourhood socioeconomic disadvantage and walking for transport. *Health & place* 19, 89-98.

# Supplementary Material 2: Methods used to identify dietary patterns

The AusDiab3 study participants completed a validated self-administered semi-quantitative food frequency questionnaire, which assessed the daily intake of 74 food items (on a 10-point frequency scale) over the past 12 months, with an additional 18 questions on usual eating habits, portion size, and intake of alcoholic beverages (Hodge *et al*. 2000). These 102 items were classified into the following 35 groups:

| **Group #** | **Name** | **Group #** | **Name** | **Group #** | **Name** |
| --- | --- | --- | --- | --- | --- |
| 1 | High fat dairy | 13 | Other cereals | 25 | Eggs |
| 2 | Medium fat dairy | 14 | Potatoes | 26 | Unsaturated spread |
| 3 | Skim milk | 15 | Citrus fruit | 27 | Saturated spread |
| 4 | Flavored milk | 16 | Other fruit | 28 | Beer |
| 5 | Juices | 17 | Fruity vegetables | 29 | Wine |
| 6 | Red Meat | 18 | Stalk vegetables | 30 | Spirits |
| 7 | Processed meat | 19 | Root vegetables | 31 | Snacks |
| 8 | Poultry | 20 | Cabbages | 32 | Nuts |
| 9 | Fish | 21 | Leafy vegetables | 33 | Peanut butter |
| 10 | Takeaway foods | 22 | Legumes | 34 | Jam vegemite |
| 11 | Pasta and rice | 23 | High fibre bread | 35 | Tomato sauce |
| 12 | High fiber cereal | 24 | White bread |  |  |

We used factor analysis (principal component method) with varimax rotation to identify the underlying dietary pattern factors associated with these 35 food groups. The final number of factors was determined by assessing eigenvalue >1, scree plot, and interpretability of the factors. Three major dietary pattern factors were identified. Factor loadings of each of these are shown in Figure S2. These diet patterns were named as: prudent, Western, and mixed. They explained 9.2%, 8.9%, and 8.5% of the variance in food intake items, respectively. Western diet was characterised by high consumption of take-away foods, snacks, processed meat, and red meat. Mixed diet was characterised by high consumption of fish, cereals, pasta, rice, and poultry. Prudent diet was characterised by high consumption of vegetables (fruity, root, leafy and stalk) and fruits.


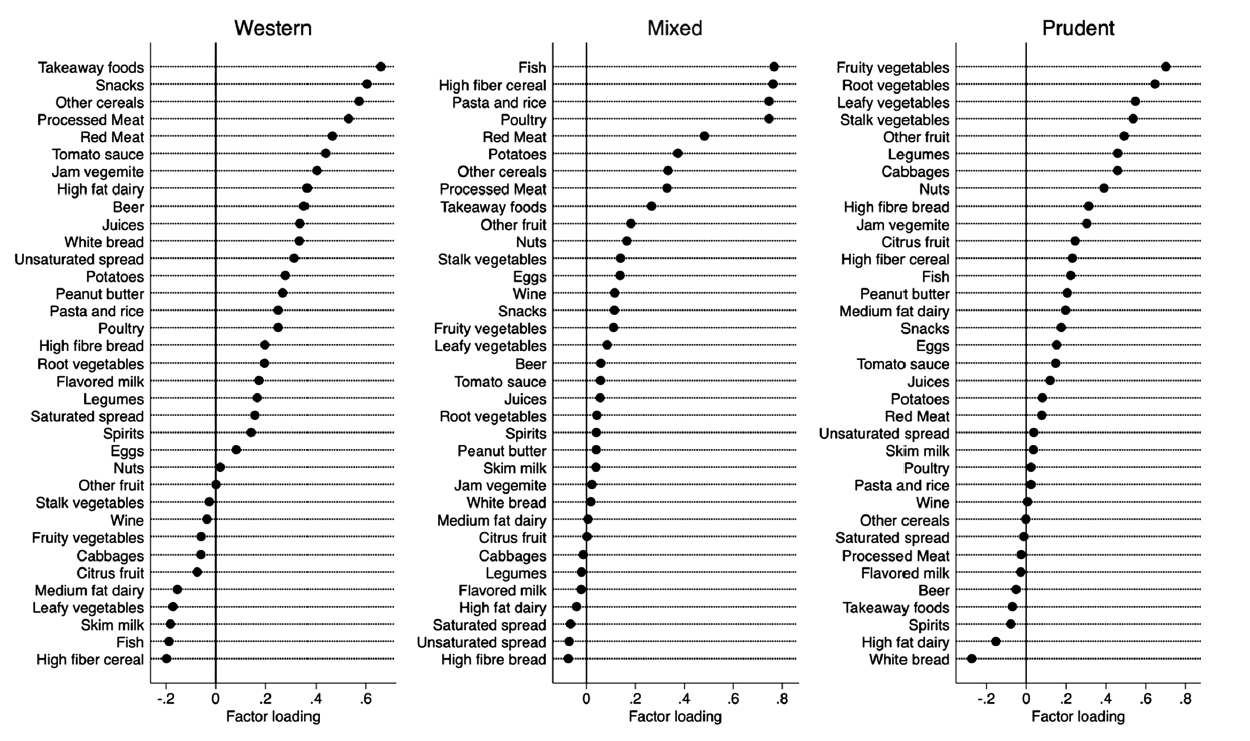


**Figure S2: Factor loadings of the three major dietary patterns based on 35 food groups. Adapted from Cao *et al* (2021).**

Reference:

Cao Y, Dunstan DW, Sethi P *et al.* (2021) The association of TV viewing time with 2-hour plasma glucose is modified by a prudent dietary pattern. *J Diabetes* 13, 661-671.

Hodge A, Patterson AJ, Brown WJ *et al.* (2000) The Anti Cancer Council of Victoria FFQ: relative validity of nutrient intakes compared with weighed food records in young to middle-aged women in a study of iron supplementation. *Aust N Z J Public Health* 24, 576-583.
